# A German translation and validation of the sense of agency scale

**DOI:** 10.3389/fpsyg.2023.1199648

**Published:** 2023-09-14

**Authors:** Victoria K. E. Bart, Dorit Wenke, Martina Rieger

**Affiliations:** ^1^Department of Psychology and Sports Medicine, Institute of Psychology, UMIT TIROL–Private University for Health Sciences and Health Technology, Hall in Tirol, Austria; ^2^Department of Psychology, PFH Private University of Applied Sciences, Göttingen, Germany

**Keywords:** sense of agency, general agency beliefs, questionnaire validation, German translation, test–retest reliability

## Abstract

Sense of agency refers to the experience of controlling one’s actions and through them events in the outside world. General agency beliefs can be measured with the Sense of Agency Scale (SoAS), which consists of the sense of positive agency subscale (i.e., feeling of being in control over one’s own body, mind, and environment) and the sense of negative agency subscale (i.e., feeling existentially helpless). The aim of the present study was to validate a German version of the SoAS. Using factor analyzes, we replicated the two-factor structure of the original version of the SoAS. Further, the German SoAS showed good model fits, good internal consistency, and moderate test–retest reliability. Construct validity was supported by significant low to moderate correlations of the German SoAS with other conceptually similar, but still distinct constructs such as general self-efficacy. Additionally, the German SoAS has an incremental value in explaining variance in the extent of subclinical symptoms of schizotypal personality disorder that goes beyond variance explained by constructs that are conceptually similar to sense of agency. Taken together, the results indicate that the German SoAS is a valid and suitable instrument to assess one’s general agency beliefs.

## Introduction

Imagine an average working day. Starting from switching on the coffee machine in the morning to switching off the light in the evening, one performs a variety of different actions that are usually followed by effects in the environment (e.g., switching on the coffee machine leads to heating up the water and eventually results in one’s morning coffee). While performing such actions one usually experiences sense of agency, i.e., the experience of controlling one’s actions and through them events in the outside world (Haggard and Tsakiris, 2009). Sense of agency is related to the awareness of one’s own mental states like perceptions, emotions, and attitudes (Newen and Vogeley, 2003), and to feelings of responsibility (Frith, 2014). Additionally, it plays an important role for well-being and mental health (*cf.* Moore, 2016; Renes and Aarts, 2017). For instance, some mental disorders such as obsessive-compulsive disorder (Belayachi and Van der Linden, 2010) and schizophrenia (Maeda et al., 2012) are associated with aberrant or distorted experiences of agency. Accordingly, assessing a person’s sense of agency may be relevant for various areas such as psychological research, diagnostics, or therapeutical settings. Whereas there exists a range of methods to assess situation-specific sense of agency, Tapal et al. (2017) were one of the first to develop a questionnaire (the Sense of Agency Scale, SoAS) that measures a person’s general, context-free agency beliefs, also sometimes termed chronic sense of agency. However, so far, the SoAS only exists in a Hebrew (Tapal et al., 2017) and French (Hurault et al., 2020) version. Thus, the aim of the present study was to provide a validated German version of the SoAS.

As the definition of sense of agency refers to the experience of control in the context of one’s own actions and their corresponding effects, in most research situation-specific sense of agency is assessed. Typically, in an experimental context, participants perform actions that are followed by certain effects. Sense of agency can be measured directly, for instance by asking participants to indicate how much control they experienced over the effect (e.g., Haering and Kiesel, 2015), how sure they are that their action caused the effect (e.g., Bart et al., 2019), or whether they feel responsible for the effect (e.g., Obhi and Hall, 2011). Sense of agency can be also measured indirectly, for instance via intentional binding (Haggard et al., 2002). This is a temporal illusion, which consists in the perception that the interval between an action and its effect is shorter if the experienced agency over the action or effect is higher compared to when it is lower (Moore and Obhi, 2012, but see Buehner, 2012). In an intentional binding task, participants are, for instance, asked to judge the timing of the action/effect (Haggard et al., 2002) or to estimate the action-effect interval (Engbert et al., 2008). By using such direct and indirect measures for situation-specific sense of agency, it has been observed that sense of agency is affected by certain situational factors such as action complexity (e.g., single-step vs. multi-step actions, Garrido-Vásquez and Rock, 2020), action selection (e.g., fluency of action selection, Wenke et al., 2010; free-choice vs. forced-choice actions, Schwarz et al., 2019), or effect type (e.g., visual vs. auditory effects, Ruess et al., 2018; positive vs. negative effects, Yoshie and Haggard, 2013).

However, it has been proposed that apart from situational variance in sense of agency, people may also have general, de-contextualized, cross-situational beliefs about having agency, i.e., beliefs regarding how much they are in control over their actions and effects in general, also sometimes termed chronic sense of agency (Tapal et al., 2017; Hurault et al., 2020). This is, for instance, supported by the observation that certain mental disorders such as depression are not only associated with distortions in situation-specific sense of agency (Scott et al., 2022), but are also associated with a general feeling of lack of control (Gask et al., 2011). One’s control beliefs or cognitions regarding agency may further affect situation-specific measures of sense of agency, particularly intentional binding. For instance, it has been observed that causal beliefs enhance intentional binding (Desantis et al., 2011; Haering and Kiesel, 2012), whereas disbelief in free will reduces intentional binding (Lynn et al., 2014). Further, in the context of an aircraft navigation task, a higher perceived extent of manual control over the navigation system (which may be akin to stronger control beliefs) enhances intentional binding (Berberian et al., 2012). Thus, sense of agency may take on different forms (situation-specific sense of agency and general agency beliefs). Whereas a range of methods to assess situation-specific sense of agency exists, the measurement of general agency beliefs is limited. The possibility to measure general agency beliefs may however help to explore the relationship between different forms of sense of agency (*cf.* Tapal et al., 2017).

So far, only a few instruments exist to measure general agency beliefs. One of them is the Sense of Agency Rating Scale (Polito et al., 2013), which was however designed to measure alterations in general agency beliefs during hypnosis and thus is not suitable to assess cross-situational sense of agency. Another one is the subjective personal agency scale (Yamaguchi et al., 2020), which was however designed to measure sense of agency in people with serious mental disorders, particularly in people suffering from schizophrenia. A further instrument is the sense of agency scale by Asai et al. (2009), which however is published in a Japanese written article and therefore the content of the article, containing not only the items of the scale itself, but also its specific theoretical focus and psychometric properties as well as the methodology used for questionnaire validation is not available for a broad readership. Recently, Tapal et al. (2017) developed the Sense of Agency Scale (SoAS) that is specifically designed to measure de-contextualized, cross-situational, general agency beliefs.

For the SoAS, in a first step, Tapal et al. (2017) reviewed relevant psychological literature on the construct of sense of agency with the aim to create items that describe the phenomenological, cognitive, and metacognitive experience of agency. This resulted in originally 36 items (worded in English), which captured multiple aspects of the agency experience such as controlling one’s action or the interaction of one’s action with the environment. The aim was to generate items that reflect one’s context-independent experience of agency and to generate items that reflect the lack thereof. In a second step, this initial set of items was then translated into Hebrew and eventually reduced to 11 items (*cf.* Tapal et al., 2017). Those items are rated on a seven-point rating scale from strongly disagree to strongly agree. Even though the authors originally expected a one-factor solution, model fits were best for a two-factor solution and thus items were assigned to two subscales: Sense of positive agency (SoPA) and sense of negative agency (SoNA). SoPA entails the feeling of being in control over one’s own body, mind, and environment, whereas SoNA entails the lack thereof, that is, feeling existentially helpless (Tapal et al., 2017). Those two subscales were only moderately correlated, indicating that SoPA and SoNA are two distinct factors (rather than two facets of the same constructs), which each measure a unique component of general agency beliefs. This is consistent with later studies which also observed that feelings of helplessness and feelings of control are only moderately (negatively) correlated and thus seem to tap into different processes (Karsh et al., 2018). Further theoretical support for the distinction between SoPA and SoNA is provided by studies showing that the feeling of being an agent (which may be akin to positive agency) and the feeling of not being an agent/someone else is the agent (which may be akin to negative agency) may be associated with activation in different brain regions (Farrer and Frith, 2002; Farrer et al., 2003; see also Tapal et al., 2017 for an extensive discussion on this topic).

The Hebrew version of the SoAS showed good psychometric properties and a good two-month test-rest reliability (see Table 1 for exact values regarding internal consistency and test–retest reliability). Further, it demonstrated good construct validity as well as incremental validity indicating that it indeed measures cross-situational, general agency beliefs. Evidence for construct validity was provided by low to moderate correlations between the SoAS subscales and other conceptually similar, but still distinct constructs (see Table 2; Tapal et al., 2017). For instance, general agency beliefs measured with the SoAS, are conceptually distinct from general self-efficacy (i.e., belief in one’s competence to successfully master difficult situations, Bandura, 1977, 1982) and physical self-efficacy (i.e., belief in one’s personal competence specifically related to one’s physical body, Ryckman et al., 1982). This demonstrates that in contrast to self-efficacy (which can be seen as one’s agency over goal related actions), the SoAS captures aspects of agency that are dissociated from goal-relevance/goal attainment. Further, Tapal et al. (2017) showed that general agency beliefs go beyond locus of control (Rotter, 1966), which is the extent to which people believe that one is in control or not in control of obtaining desired outcomes/rewards. Additionally, Tapal et al. (2017) provided evidence that the SoAS measures one’s own agency beliefs rather than culturally transmitted perceptions of the philosophical notions of free will, unpredictability and/or determinism. Moreover, based on low correlations of the SoAS with body consciousness (i.e., monitoring bodily states and bodily awareness), the authors argued that the SoAS is conceptually distinct from one’s sense of body ownership (that is, the feeling that this body/body part is one’s own, Gallagher, 2000).

**Table 1:** Overview of the internal consistency (McDonald’s ω) and two-month test–retest reliability of the Hebrew (*cf.* Tapal et al., 2017) and French (*cf.* Hurault et al., 2020) version of the Sense of Agency Scale (SoAS), separately for the sense of positive agency subscale (SoPA) and for the sense of negative agency subscale (SoNA).

	Hebrew SoAS	French SoAS
Internal consistency		
SoPA	0.80	0.65
SoNA	0.75	0.53
Test–retest reliability		
SoPA	LC: *r* = 0.78	ICC = 0.78
SoNA	LC: *r* = 0.74	ICC = 0.72

ICC, intraclass correlation coefficient; LC, latent correlation.

**Table 2:** Overview of the validity of the Hebrew version of the sense of agency scale (Tapal et al., 2017).

	SoPA	SoNA
**Construct validity**		
*External locus of control* (Rotter, 1966, higher values indicate higher external locus of control)	−0.35*	0.33*
*General self-efficacy* (Bosscher and Smit, 1998)		
Initiative (higher values indicate a lack of initiative)	−0.27*	0.35
Effort (higher values indicate willingness to expand effort)	0.43*	−0.31*
Persistence (higher values indicate less persistence)	−0.31*	0.34*
*Free-Will and Determinism Belief Scale* (Paulhus and Carey, 2011)		
Free Will (higher values indicate higher free will)	0.49*	−0.26*
Fatalistic Determinism (higher values indicate stronger belief in fate)	0.15	0.10
Scientific Determinism (higher values indicate stronger beliefs in environmental or biological forces)	−0.07	0.35*
Unpredictability (higher values indicate stronger beliefs in unpredictability)	0.12	0.24*
*Body Consciousness* (Miller et al., 1981)		
Private Body Consciousness (higher values indicate higher awareness of internal sensations)	0.02	0.08
Public Body Consciousness (higher values indicate higher awareness of observable aspects of the body)	0.09	0.06
Body Competence (higher values indicate higher perceived physical competence)	0.24*	−0.10
*Physical self-efficacy* (Ryckman et al., 1982)		
Ability (higher values indicate higher perceived competence in performing tasks involving the use of physical skills)	0.16*	−0.06
Self-presentation Confidence (higher values indicate higher confidence in displaying physical skills and having them evaluated by others)	0.45*	−0.45*
**Incremental validity**		
*Depression* (Beck depression Inventory-II, Beck et al., 1996)	−0.01	0.03
*Obsessive compulsive disorder* (Obsessive-Compulsive Inventory Revised, Foa et al., 2002)	−0.11	0.35*

*Significant at *p* < 0.05. The upper part of the table provides information regarding its construct validity [latent correlations of the sense of positive agency subscale (SoPA) and the sense of negative agency subscale (SoNA) with constructs that are conceptually similar, but still distinct from sense of agency]. The lower part of the table provides information regarding its incremental validity (latent correlations of the two sense of agency subscales with subclinical symptoms of depression and obsessive-compulsive disorder, after controlling for all of the other constructs).

Further, Tapal et al. (2017) examined whether the SoAS has an incremental value over and above conceptually similar constructs. It has been proposed that certain mental disorders are associated with distortions in one’s sense of agency. For instance, sense of agency seems to be associated with obsessive-compulsive disorder (see Szalai, 2019 for a review) and depression (Stephan, 2013). Conceptually similar constructs to sense of agency such as locus of control and general self-efficacy are related to mental health issues (Kim, 2003) and are related to the above-mentioned disorders (e.g., Benassi et al., 1988; Kennedy et al., 1998; Kalafat et al., 2010). This may not only be the case in clinical samples but may hold also true for subclinical manifestations of those disorders in healthy samples. Tapal et al. (2017) showed that even though the SoAS does not explain variance of depressive symptoms that goes beyond variance explained by conceptually similar constructs (self-efficacy and locus of control), it does explain additional variance of symptoms of obsessive-compulsive disorder, confirming incremental validity of the SoAS (see Table 2 for exact values).

Taken together, this may indicate that there is indeed the need for an instrument that covers general agency beliefs and research on sense of agency may benefit from the possibility to not only measure contextualized, situation-specific sense of agency but also a more general, de-contextualized component of sense of agency. However, so far validated versions of the SoAS are only available in the original Hebrew version (Tapal et al., 2017) and a recently translated French version (Hurault et al., 2020, see Table 1 for internal consistency and test–retest reliability of this version).

Thus, the aim of the present study was to provide a validated, German version of the SoAS such that the measurement of general agency beliefs becomes also available for German-speaking countries. In particular, we first aimed to replicate the two-factorial structure obtained in the Hebrew and French version of the SoAS for our German version in a sample of German-speaking participants. Further, we aimed to assess the construct validity of the German SoAS by investigating the relationship between general agency beliefs (assessed via the SoAS) and other conceptually similar, but still distinct constructs. To this end we assessed the same constructs as Tapal et al. (2017), which were general self-efficacy, locus of control, belief in free will/determinism, and body consciousness. In the present study, body consciousness was assessed via interoceptive awareness, i.e., the conscious perception of sensations from inside the body (Vaitl, 1996; Mehling et al., 2012) and body image, i.e., the perception of one’s appearance and well-being as well as the perception of one’s vitality and energy (Clement and Löwe, 1996; Albani et al., 2006).^1^ In contrast to Tapal et al. (2017) we did not assess physical self-efficacy because some of those items are quite similar to items assessing interoceptive awareness.

We also aimed to assess the incremental validity of the German SoAS. Thus, similar to Tapal et al. (2017), we investigated whether the German SoAS has any incremental value in explaining variance in the extent of certain subclinical symptoms beyond conceptually similar constructs. In addition to obsessive-compulsive disorder and depressive disorder (as used by Tapal et al., 2017), we also assessed incremental validity for explaining subclinical symptoms of schizotypal personality disorder because this disorder also seems to be associated with sense of agency (Asai and Tanno, 2007, 2008; Moore et al., 2021). Last but not least, we aimed to assess test–retest reliability of the German SoAS after a time period of 2 month.

## Method

### Participants

Five hundred seventeen German-speaking participants, which included psychology students that received course credit for their participation as well as participants recruited through acquaintances and through social media, took part in the study (age: *M* = 28.47, *SD = 10.48; sex: 335 female, 179 male, 2 diverse, 1 did not wish to say; country of living:* 279 from Austria, 84 from Germany, 153 from South Tyrol in Italy, 1 from Switzerland; level of education: 121 had a university degree, 270 had completed a high school or trade school with higher education entrance qualification, 82 had completed a high school or trade school without higher education entrance qualification, 40 had completed compulsory school, 2 had not completed school, 2 did not wish to say). To assess test–retest reliability we asked participants whether they would be willing to participate in a follow up survey. Two Hundred and Thirty Five participants agreed and were thus contacted again after a period of 2 months. Eighty Six of those participants (that is 16.63% of the total sample, age: *M* = 28.69, SD = 10.75 sex: 62 female, 24 male) followed the invitation and completed the SoAS again.

The required sample size was estimated based on previous studies, which already have developed and validated different versions of the SoAS (see Tapal et al., 2017 for the original Hebrew version; Hurault et al., 2020 for the French adaption) as well as based on recommendations in the literature suggesting a participant to item ratio of 20:1 for exploratory factor analyzes (Costello and Osborne, 2005) and a minimum of 200 participants for confirmatory factor analyzes (for a two-factor model with factor-loadings of 0.5 and about 6 items per factor, Wolf et al., 2013).

All procedures in the present study were in accordance with the 1964 Helsinki declaration and its later amendments. The study was approved by the local ethics committee and participants gave informed consent.

### Material and procedure

Upon requesting approval of the authors to translate their questionnaire, we used their English translation of the Hebrew items (Tapal et al., 2017) and translated the 11 English items into German. To this end, we combined a backtranslation approach (*cf.* Brislin, 1970; Bracken and Barona, 1991; Beaton et al., 2000; International Test Commission, 2017) and an interactive translation approach (*cf.* Douglas and Craig, 2007). First, two independent German translations were prepared by native German speakers, who were also proficient in English. Differences in those two versions were discussed until a consensus was reached. The final version was then back translated into English by a third independent translator who was blinded to the initial English questionnaire. The original and back translated versions were compared, and discrepancies were discussed by experts in the field resulting in a unified German version. In a final step, some participants (*N* = 8) were asked to provide feedback about their understanding of the meaning of questions, the ease of comprehension, and clarity of the questions, which was again discussed by experts in the field resulting in the final German translation (*cf.* Douglas and Craig, 2007). The German version of the SoAS is available at the open science framework, https://osf.io/muhzr/ (and in the Appendix). The *SoAS* consists of the SoPA (i.e., feeling in control, 5 items) and the SoNA (i.e., existentially feeling helpless, 6 items) subscales (Tapal et al., 2017). Each item was answered on a seven-point rating scale, ranging from strongly disagree (1) to strongly agree (7).

We used additional questionnaires to assess further constructs that may be related to sense of agency. *Locus of control* was assessed with the Scale for Internal-External Locus of Control-4 (Kovaleva et al., 2014), which consists of two subscales (internal locus of control: 2 items, McDonald’s ω ranging between 0.70 and 0.71; external locus of control: 2 items, McDonald’s ω ranging between 0.53 and 0.63). Items were answered on a five-point rating sale from not at all true (1) to exactly true (5). *General self-efficacy* was assessed with the general self-efficacy scale (Schwarzer and Jerusalem, 1999, Cronbach’s α ranging between 0.80 and 0.90), which consists of 10 items that were answered on a four-point rating scale from not at all true (1) to exactly true (4). *Belief in free will* was assessed with the Belief in Free Will Inventory (Melcher, 2019), which consists of five subscales (situational determinism: 5 items, Cronbach’s *α* = 0.80; free will: 5 items, Cronbach’s *α* = 0.78; indeterminism/chance: 4 items, Cronbach’s *α*: = 0.74; biological determinism: 4 Items, Cronbach’s *α* = 0.69; incompatibilism: 3 Items, Cronbach’s *α* = 0.69). Items were answered on a five-point rating scale from strongly disagree (1) to strongly agree (5). *Interoceptive awareness* was assessed with the Multidimensional Assessment of Interoceptive Awareness (Mehling et al., 2012; see Bornemann et al., 2015 for a German version), which consists of 8 subscales (noticing: 4 items, Cronbach’s *α* = 0.76; not-distracting: 3 items, Cronbach’s *α* = 0.56; not-worrying: 3 items, Cronbach’s *α* = 0.65; attention regulation: 7 items, Cronbach’s *α* = 0.89; emotional awareness: 5 items, Cronbach’s *α* = 0.86; self-regulation: 4 items, Cronbach’s *α* = 0.84; body listening: 3 items, Cronbach’s *α* = 0.84; trusting: 3 items, Cronbach’s *α* = 0.86). Items were answered on a six-point rating scale from never (0) to always (5). *Body image* was assessed with the body image questionnaire (Clement and Löwe, 1996; Albani et al., 2006), which consists of two subscales (rejecting body evaluation: 10 items, Cronbach’s *α* = 0.84; vital body dynamics: 10 items, Cronbach’s *α* = 0.91). Items were answered on a five-point rating scale from not at all true (1) to completely true (5).

Further we used questionnaires to assess the extent of subclinical symptoms of certain mental disorders. The extent of subclinical symptoms of the *obsessive-compulsive disorder* was assessed with the Obsessive-Compulsive Inventory Revised (Foa et al., 2002; see Gönner et al., 2007 for a German version, Cronbach’s α over all subscales = 0.85), which consists of six subscales (washing, obsessing, hoarding, ordering, checking, neutralizing) with three items each that describe different kinds of experiences. Participants were asked to indicate on a five-point rating scale from not at all (0) to extremely (4) how much those experiences have distressed or bothered them during the past month. The extent of subclinical symptoms of *depression* was assessed with the Beck-Depression Inventory-II (Beck et al., 1996; see Hautzinger et al., 2009 for a German version, Cronbach’s *α* = 0.90) consisting of 21 items that relate to different symptoms of depression. Each item entails four statements and participants were asked to choose the statement that describes best how they have been feeling throughout the past 2 weeks. The extent of subclinical symptoms of the *schizotypal personality disorder* was assessed with the Schizotypal Personality Questionnaire (Raine, 1991; see Klein et al., 1997 for a German version, Cronbach’s α over all subscales = 0.88) which consists of nine subscales (ideas of reference: 9 items; excessive social anxiety: 8 items; odd beliefs/magical thinking: 7 items; unusual perceptual experiences: 9 items; odd or eccentric behavior: 7 items; no close friends: 9 items; odd speech: 9 items, constricted affect: 8 items, suspiciousness: 8 items). Items entail certain opinions, experiences or patterns of behavior and participants were asked to indicate with no or yes whether they agree to them or not.

The study was administered online using Lime Survey (version 3.15.0). Participants first completed the German version of the SoAS. Afterwards they completed the remaining questionnaires in random order. At the end, participants were asked whether they would be willing to participate again. If so, they were contacted 2 months after their first participation and were asked to complete the German version of the SoAS again.

### Data analysis

Data are available at the Open Science Framework, https://osf.io/muhzr/. To examine the psychometric properties of the German SoAS, we used a two-step analytic strategy, i.e., we performed an exploratory factor analysis followed by a confirmatory factor analysis (*cf.* Swami and Barron, 2019, who recommend this approach for questionnaire translations). Following a cross-validation approach (*cf.* Cudeck and Browne, 1983; Anderson and Gerbing, 1988; see also Fokkema and Greiff, 2017), we randomly split the sample into two independent samples. With the first sample (*N* = 258) we performed an exploratory factor analysis to assess the underlying factor structure of the German SoAS. This enabled us to assess the dimensionality of the translated questionnaire without any a priori limitations in terms of modeling. With the second sample (*N* = 259) we performed a confirmatory factor analysis to determine how well the model derived by the exploratory factor analysis fits the data. The complete sample (*N* = 517) was used to assess construct validity and incremental validity. Data from a subsample of it (*N* = 86), who followed our invitation to complete the SoAS again 2 months after initially taking part in the study, were used to assess test–retest reliability. For details on the respective analyzes see the results section.

## Results

### Exploratory factor analysis

On a random sample of half of the data an exploratory factor analysis was computed using jamovi, version 2.2.5 (The jamovi project, 2021).^2^ The Kaiser-Meyer-Olkin (KMO) test of sampling adequacy and Bartlett’s test of sphericity were used to determine the suitability of the data for factor analysis. An overall KMO value of 0.84 confirmed sampling adequacy and all KMO values for the individual items were above the acceptable limit of 0.50 (Kaiser, 1974). Additionally, Bartlett’s test of sphericity, *χ*^2^ (55) = 938.45, *p* < 0.001, indicated that correlations between the items were sufficiently large to perform an exploratory factor analysis. For the EFA, principal axis as extraction method was used because Mardia’s tests for skewness and kurtosis (*p*_max_ < 0.001, conducted using WebPower, Zhang and Yuan, 2018)^3^ indicated deviations from multivariate normality (Mardia, 1970). Further, oblique rotation (oblimin) was used because we assumed that the underlying factors are correlated (as has been observed in the Hebrew and French version of the SoAS, Tapal et al., 2017; Hurault et al., 2020).

**Figure 1:**
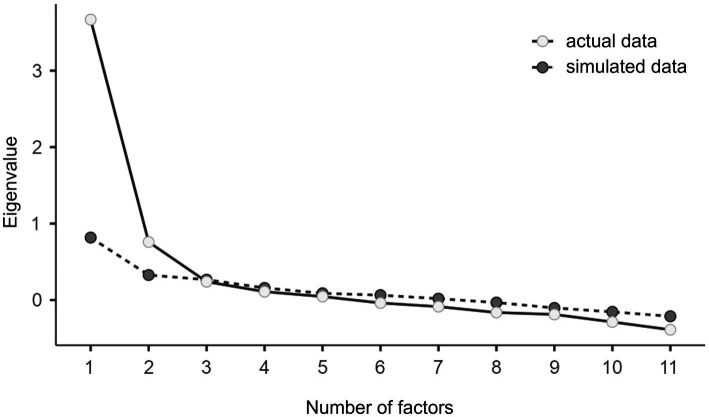
Screeplot with the number of factors on the x-axis and the eigenvalues on the y-axis for the actual and simulated data. For factor 1 and factor 2 the eigenvalues of the actual data are higher than the eigenvalues of the simulated data resulting in a two-factor solution.

The number of factors was determined based on parallel analysis. That is, eigenvalues in the actual data set are compared with estimated eigenvalues from a simulated random data set. Only factors with eigenvalues larger than those in the simulated random data set are extracted (Horn, 1965). Based on this criterion, a two-factor solution was chosen (see Figure 1). Table 3 shows the factor loadings for the two-factor model after rotation. Considering the content of the items loading on the respective factors, the two factors corresponded well to the SoPA and SoNA factor proposed in previous studies (Tapal et al., 2017; Hurault et al., 2020).

**Table 3:** Factor loadings of the items after rotation for the sense of positive agency (SoPA) and the sense of negative agency (SoNA) factor.

Item in English	Item in German	SoPA	SoNA
1. I am in full control of what I do.	1. Ich habe die volle Kontrolle darüber, was ich tue.	**0.47**	−0.25
2. I am just an instrument in the hands of somebody or something else.	2. Ich bin nur ein Instrument in den Händen von jemandem oder von etwas anderem.	−0.16	**0.56**
3. My actions just happen without my intention.	3. Meine Handlungen geschehen einfach ohne meine Absicht.	−0.04	**0.78**
4. My movements are automatic–my body simply makes them.	4. Meine Bewegungen geschehen automatisch - mein Körper macht sie einfach.	0.07	**0.32**
5. The outcomes of my actions generally surprise me.	5. Im Allgemeinen überraschen mich die Ergebnisse meiner Handlungen.	0.03	**0.69**
6. Things I do are subject only to my free will.	6. Sachen, die ich mache, unterliegen nur meinem freien Willen.	**0.69**	0.05
7. The decision whether and when to act is within my hands.	7. Die Entscheidung, ob und wann ich handle, liegt in meinen Händen.	**0.69**	−0.12
8. Nothing I do is actually voluntary.	8. Nichts was ich mache, ist wirklich freiwillig.	0.003	**0.69**
9. While I am in action, I feel like I am a remote controlled robot.	9. Während ich handle, fühle ich mich wie ein ferngesteuerter Roboter.	0.02	**0.83**
10. My behavior is planned by me from the very beginning to the very end.	10. Mein Verhalten ist von mir von Anfang bis Ende durchgeplant.	**0.51**	0.24
11. I am completely responsible for everything that results from my actions.	11. Ich bin vollkommen verantwortlich für alles, was sich aus meinen Handlungen ergibt.	**0.47**	−0.08

The higher of the two factor loadings is depicted in bold.

The two factors were negatively correlated (*r* = − 0.54) and explained a total of 42.18% of the variance (SoPA: 16.03%, SoNA: 26.15%). The internal consistency was measured using McDonald’s ω, which was 0.72 for SoPA and 0.82 for SoNA. After excluding item 4 from the SoNA subscale (see below), McDonald’s ω changed to 0.85.

All items, except for one item (item 4: My movements are automatic–my body simply makes them), fulfilled the item inclusion criteria, which are (a) loading above 0.40 onto the primary factor, (b) loading below 0.30 onto the alternative factors, and (c) a difference of 0.20 between primary and alternative factor loadings (Howard, 2016). Thus, item 4 cannot be unambiguously assigned to one factor and was therefore removed from the following analyzes (confirmatory factor analysis as well as the analysis regarding validity and re-test reliability).

### Confirmatory factor analysis

On the other half of the data a confirmatory factor analysis was computed using R (R Core Team, 2021)^4^ within the RStudio environment to test whether the model identified with the exploratory factor analysis fits the data adequately. Mardia’s tests for skewness and kurtosis again indicated deviations from multivariate normality (*p*_max_ < 0.001, conducted using WebPower) (see footnote 3). Thus, we used maximum likelihood robust as estimator. The variances of both latent factors were fixed at 1. The following fit indices were conducted: Chi-squared statistic (*χ*^2^), Comparative Fit Index (CFI), Standardized Root Mean Square Residual (SRMR), and Root Mean Square Error of Approximation (RMSEA). A ratio of the chi-square statistic to the respective degrees of freedom (*χ*^2^/df) of less than 2, a CFI greater than 0.95, a SRMR of less than 0.08, and a RMSEA of less than 0.06 indicate a good fit (Hu and Bentler, 1999; Schreiber et al., 2006).

**Figure 2:**
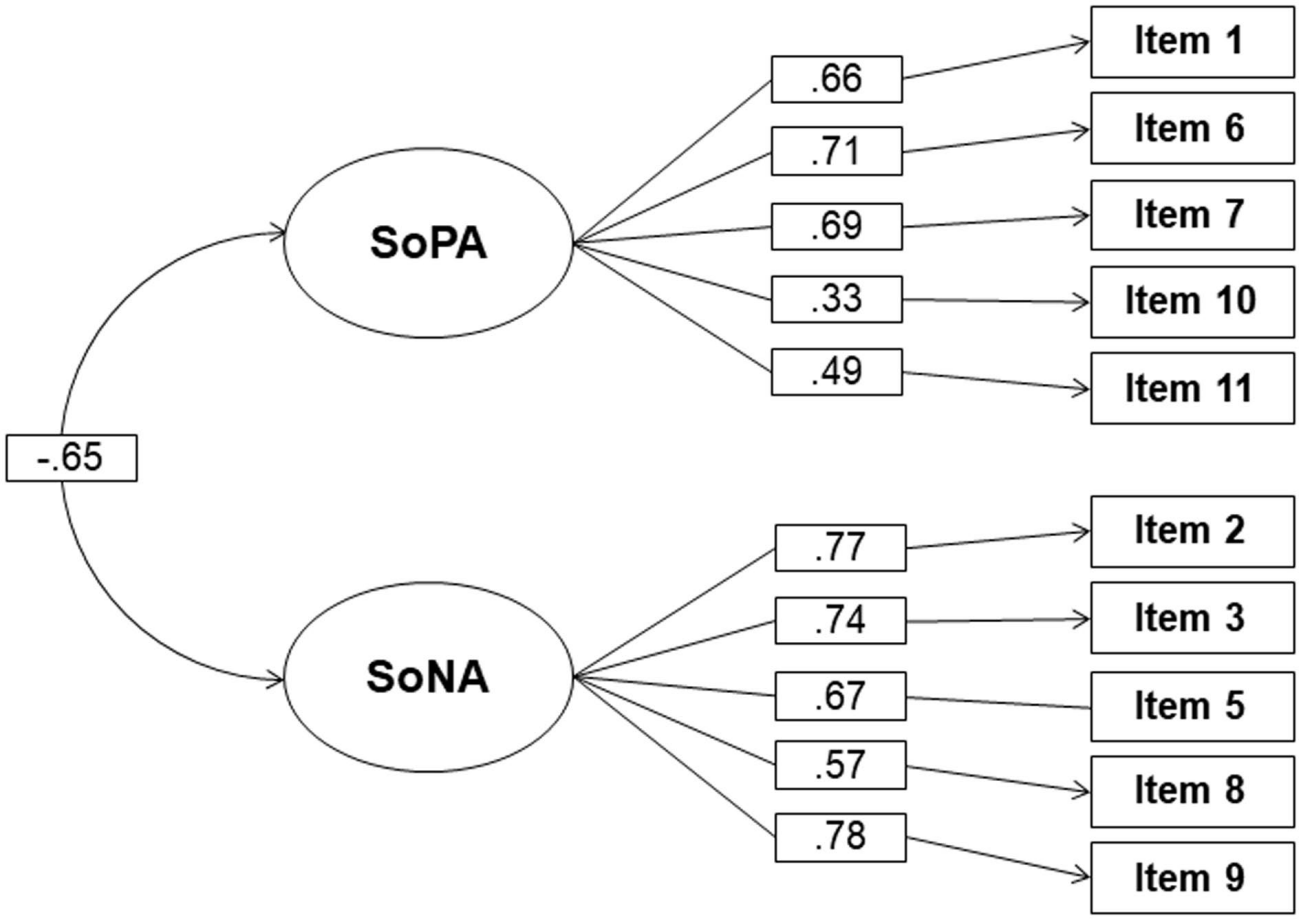
Path diagram showing the latent factor covariance and the standardized factor loadings of each item for the two-factor confirmatory model with the factors sense of positive agency (SoPA) and sense of negative agency (SoNA).

The model diagram with the parameter estimates can be seen in Figure 2. The model fitted the data adequately with *χ*^2^(34) = 44.52, *p* = 0.107, *χ*^2^/df = 1.31, *CFI* = 0.98, *SRMR* = 0.05, and *RMSEA* = 0.04. The covariance between the two factors was −0.65 (*p* < 0.001). The internal consistency was again measured using McDonald’s ω, which was 0.72 for SoPA and 0.83 for SoNA.

As one may question whether SoPA and SoNA are indeed two distinct factors or rather two facets of the same construct (see Tapal et al., 2017 for such a discussion), we also ran a one-factor model confirmatory factor analysis. However, the fit indices, *χ*^2^(35) = 106.44, *p* < 0.001, *χ*^2^/df = 3.04, CFI = 0.88, SRMR = 0.08, and RMSEA = 0.10, indicated a rather poor model fit of the one-factor model.

### Construct validity

Construct validity was assessed using jamovi, version 2.2.5 (The jamovi project, 2021) (see footnote 2) by conducting Pearson correlations between the mean scores of the two subscales of the SoAS (SoPA: *M* = 5.29, *SD* = 0.94, SoNA: *M*  = 1.95, *SD* = 1.01) and the mean scores of conceptually similar constructs: locus of control, general self-efficacy, free will, interoceptive awareness, and body image. In Table 4 the descriptive statistics of those constructs as well as the correlation results of those constructs with the SoAS subscales can be seen (for the correlations of those constructs with each other see Supplementary Tables S1, S2 in the Supplemental material).

**Table 4:** Descriptive statistics (means and standard deviations) of the mean scores of locus of control (2 subscales: internal locus of control, external locus of control), general self-efficacy, free will (5 subscales: situational determinism, free will, indeterminism/change, biological determinism, incompatibilism), interoceptive awareness (8 subscales: noticing, not-distracting, not-worrying, attention regulation, emotional awareness, self-regulation, body listening, trusting), and body image (2 subscales: rejecting body evaluation, vital body dynamics). Further, results of Pearson correlations between the mean scores of those constructs and the mean scores of the sense of positive agency (SoPA) and sense of negative agency (SoNA) subscales are depicted.

		SoPA		SoNA	
	*M* (SD)	*r*	*p*	*r*	*p*
**Locus of control**					
Internal locus of control	4.28 (0.67)	0.44	<0.001	- 0.32	<0.001
External locus of control	2.33 (0.85)	- 0.36	<0.001	0.51	<0.001
General self-efficacy	2.99 (0.47)	0.32	<0.001	- 0.32	<0.001
**Belief in free will**					
Situational determinism	3.04 (0.74)	0.16	<0.001	0.05	0.220
Free will	3.86 (0.68)	0.31	<0.001	- 0.42	<0.001
Indeterminism/change	2.85 (0.95)	- 0.09	0.038	0.15	<0.001
Biological determinism	3.02 (0.68)	0.06	0.184	0.06	0.154
Incompatibilism	2.94 (0.94)	0.04	0.313	0.06	0.170
**Interoceptive awareness**					
Noticing	3.34 (0.96)	0.10	0.020	- 0.16	<0.001
Not-distracting	2.13 (0.98)	- 0.02	0.622	−0.07	0.097
Not-worrying	2.53 (0.91)	0.06	0.160	- 0.10	0.025
Attention regulation	2.92 (0.94)	0.21	<0.001	- 0.12	0.009
Emotional awareness	3.55 (1.02)	0.17	<0.001	- 0.14	0.002
Self-regulation	2.78 (1.10)	0.14	0.001	- 0.09	0.048
Body listening	2.51 (1.20)	0.09	0.049	0.04	0.415
Trusting	3.65 (1.14)	0.29	<0.001	- 0.27	<0.001
**Body image**					
Rejecting body evaluation	2.19 (0.77)	- 0.30	<0.001	0.42	<0.001
Vital body dynamics	3.49 (0.61)	0.25	<0.001	- 0.26	<0.001

We mostly observed significant low to moderate correlations between the different constructs, indicating that the SoAS subscales are related to conceptually similar constructs, but are still conceptually distinct enough to warrant their uniqueness, thereby supporting construct validity of the German SoAS.

### Incremental validity

It was examined whether the SoAS has any incremental value in explaining variance in the extent of subclinical symptoms of certain mental disorders, that goes beyond variance explained by constructs that are conceptually similar to sense of agency. To this end, IBM SPSS Statistics (Version 27) was used to run three identical hierarchical linear regression analyzes with the sum scores of the Obsessive-Compulsive Inventory Revised (sum score was calculated over all six subscales, see Gönner et al., 2007, *M* = 18.13, *SD* = 11.99), the Beck Depression Inventory-II (*M* = 8.63, *SD* = 8.73), and the Schizotypal Personality Questionnaire (sum score was calculated over all 9 subscales, see Klein et al., 1997, *M* = 16.54, *SD* = 12.01), respectively as dependent variable. In each analysis we included locus of control (internal and external subscale) and self-efficacy as predictors in Step 1. In Step 2 we entered SoPA and SoNA as additional predictors. Preliminary analyzes revealed no violation of assumptions of linearity and multicollinearity. However, the assumptions of normality and homoscedasticity were violated. Thus, coefficients were tested with HC4 robust standard errors (SE) according to the recommendation of Hayes and Cai (2007). The coefficients of those linear hierarchal regression models can be seen in Table 5.

**Table 5:** Coefficients of the frequentist linear hierarchical regression models and Bayes Inclusion factors (BF_inc_) of the Bayesian linear regression for the sum scores of the Obsessive-Compulsive Inventory Revised, the Beck Depression Inventory-II, and the Schizotypal Personality Questionnaire as dependent variables.

	*b*	*SE*	β	*p*	*BF_inc_*	*R*^2^_adj_, *p*
**Obsessive-compulsive inventory revised**						
*Step 1*						0.095, <0.001
Constant	23.34	5.54		<0.001	1	
Internal locus of control	0.04	0.93	0.002	0.962	0.15	
External locus of control	3.14	0.74	0.22	<0.001	>100	
General self-efficacy	- 4.25	1.50	- 0.17	0.005	>100	
*Step 2*						0.106, <0.001
Constant	20.57	6.43		0.001	1	
Internal locus of control	0.44	0.96	0.03	0.645	0.18	
External locus of control	2.26	0.91	0.16	0.013	26.35	
General self-efficacy	- 3.70	1.55	- 0.15	0.017	19.74	
SoPA	- 0.32	0.71	- 0.03	0.659	0.20	
SoNA	1.60	0.84	0.13	0.058	6.97	
**Beck depression inventory-II**						
*Step 1*						0.191, <0.001
Constant	29.20	4.56		<0.001	1	
Internal locus of control	- 1.54	0.70	- 0.12	0.028	3.35	
External locus of control	1.37	0.52	0.13	0.009	15.68	
General self-efficacy	- 5.75	1.16	−0.31	<0.001	>100	
*Step 2*						0.194, <0.001
Constant	32.41	4.94		<0.001	1	
Internal locus of control	- 1.50	0.71	- 0.12	0.034	3.65	
External locus of control	1.70	0.57	0.17	0.003	21.26	
General self-efficacy	- 5.91	1.12	- 0.32	<0.001	>100	
SoPA	- 0.39	0.48	- 0.04	0.421	0.19	
SoNA	- 0.84	0.57	−0.10	0.144	0.57	
Schizotypal personality questionnaire						
*Step 1*						0.062,<0.001
Constant	38.13	5.71		<0.001	1	
Internal locus of control	- 1.33	0.98	- 0.07	0.177	0.46	
External locus of control	0.18	0.79	0.01	0.816	0.16	
General self-efficacy	- 5.46	1.55	- 0.21	<0.001	>100	
*Step 2*						0.084, <0.001
Constant	45.59	6.32		<0.001	1	
Internal locus of control	- 1.46	1.01	- 0.08	0.149	1.14	
External locus of control	1.24	0.84	0.09	0.140	0.97	
General self-efficacy	- 6.02	1.43	- 0.24	<0.001	>100	
SoPA	- 0.59	0.71	- 0.05	0.410	0.36	
SoNA	- 2.37	0.78	- 0.20	0.002	20.69	

BFInclusion = model-averaged Bayes factor of model including an effect relative to model excluding it. Step 1 includes locus of control and general self-efficacy as predictors and Step 2 additionally includes the sense of positive agency (SoPA) and sense of negative agency (SoNA) subscales as predictors.

For the Obsessive-Compulsive Inventory Revised the model in Step 2, i.e., the model including the SoAS subscales, performed significantly better than the model in Step 1, *F*(2, 511) = 4.21, *p* = 0.015, ΔR^2^_adj._ = 0.011. However, at predictor level, SoPA and SoNA were not significant. As multicollinearity is not the problem here (VIFs <10), the most likely explanation for the inconsistent results is that the value of p of the SoNA predictor is close to significance. For the Beck Depression Inventory-II the models in Step 1 and Step 2 did not significantly differ from each other, *F*(2, 511) = 1.93, *p* = 0.146, ΔR^2^_adj._ = 0.003. For the Schizotypal Personality Questionnaire, the model in Step 2 performed significantly better than the model in Step 1, *F*(2, 511) = 7.08, *p* < 0.001, ΔR^2^_adj._ = 0.022, indicating that the SoAS (particularly driven by SoNA) accounted for additionally 2.20% of variance beyond locus of control and general self-efficacy.

Because there was hardly any incremental value of the SoAS in explaining variance in the extent of subclinical symptoms of obsessive-compulsive disorder and depressive disorder, and a rather low incremental value to explain subclinical symptoms of schizotypal personality disorder, we additionally ran Bayesian linear regressions using jasp., version 0.16.4 (JASP Team, 2023).^5^ This allows to quantify the evidence for or against an incremental value of the SoAS. We compared a model that includes SoPA and SoNA as predictors against a null model, which only contains locus of control (internal and external subscale) and self-efficacy as predictors. We used a uniform prior, which assumes all models are equally likely to explain the data (*cf.* Faulkenberry et al., 2020). BF_10_ greater than 3 indicate substantial evidence for the presence of an effect, BF_10_ between 1 and 3 indicate anecdotal evidence for the presence of an effect, BF_10_ between 1/3 and 1 indicate anecdotal evidence for the absence of an effect, and BF_10_ smaller than 1/3 indicate substantial evidence for the absence of an effect (Jeffreys, 1961; Jarosz and Wiley, 2014). Evidence for an incremental value of the SoAS as a predictor for subclinical symptoms of obsessive-compulsive disorder was anecdotal (BF_10_ = 1.74). However, if one only includes SoNA in the model (rather than SoNA and SoPA), evidence becomes substantial (BF_10_ = 8.48). Further, evidence for an incremental value of the SoAS as a predictor for subclinical symptoms of schizotypal personality disorder was substantial (BF_10_ = 29.12), again mainly driven by SoNA. Regarding subclinical symptoms of depressive disorder, there was substantial evidence against an incremental value of the SoAS as a predictor (BF_10_ = 0.14). Bayes factors quantifying the evidence for the inclusion of a particular predictor (averaged across all models that contain the predictor) relative to models that do not contain that particular predictor (BF_inc_) can be seen in Table 5.

Taken together, those results indicate that the German SoAS has an incremental value in explaining variance in the extent of subclinical symptoms of schizotypal personality disorder and (based on Bayesian analysis) obsessive-compulsive disorder that goes beyond variance explained by constructs that are conceptually similar to sense of agency. Thus, the results provide tentative evidence for the incremental validity of the German SoAS. However, this should be investigated more thoroughly in follow-up studies, particularly as the additionally explained variance was rather low, evidence regarding subclinical symptoms of obsessive-compulsive disorder was anecdotal, and there was no incremental effect for SoAS as predictor for subclinical symptoms of depressive disorder.

### Test–retest reliability

Test–Retest reliability was assessed by calculating intraclass correlation coefficients (ICC, average measurements, absolute-agreement, 2-way mixed-effects model, Koo and Li, 2016) between the first and the second measurement, separately for the SoPA and for the SoNA subscale using the ‘seolmatrix’ module of jamovi (see footnote 2). ICCs were 0.69 (*p* < 0.001) for SoPA and 0.70 (*p* < 0.001) for SoNA, demonstrating moderate test–retest reliability (Koo and Li, 2016).

## Discussion

The aim of the present study was to provide a validated German version of the SoAS. We replicated the two-factor structure of the original version of the SoAS for the German version. Further, the German SoAS showed good model fits, good internal consistency, and moderate test–retest reliability. We observed significant low to moderate correlations between the German SoAS and conceptually similar constructs such as general-self efficacy, locus of control and free will. Additionally, we observed that the German SoAS (in particular, the SoNA subscale) accounts for additional variance in the extent of subclinical symptoms of schizotypal personality disorder beyond variance explained by conceptually similar constructs.

An initially performed exploratory factor analysis on the German SoAS replicated the two-factor model observed in the original Hebrew version (Tapal et al., 2017) and its French translation (Hurault et al., 2020). Further, except for one item (item 4), all items showed sufficient factor loadings on their respective factor (SoPA, SoNA) corresponding to the original Hebrew version (Tapal et al., 2017). This indicates that one’s general agency beliefs indeed consist of a positive component (SoPA, feeling of being in control) and a negative component (SoNA, feeling existentially helpless) and that most of the translated items were adequately worded to capture those differences. There may be several explanations for the rather low factor loading of item 4 (“My movements are automatic–my body simply makes them.”). One explanation is that the two parts of the sentence elicit contradictory associations. Whereas the term “automatic” in the first part of the sentence refers to a total lack of control, the term “simply” in the second part of the sentence refers to an ease of control (*cf.* Hurault et al., 2020 for a similar argument for the French version of the SoAS, in which this item was also problematic). Additionally, the first part of the sentence refers to the performed action itself (i.e., the movement), whereas the second part of the sentence refers to the initiator of the action (i.e., one’s body). Those inconsistencies in item content may have affected response behavior differently, depending on which part of the sentence was more attended to. Another, maybe more likely, explanation may be that the content of item 4 is markedly different from the content of all other items of the scale. Item 4 is the only item that refers to a concrete action (bodily action), whereas all other items refer to actions in general, which may have caused different response behavior. Taken together, item 4 does not seem to be suitable to assess SoNA (at least for the German version of the SoAS). Therefore, we removed this item resulting in a 10-item version of the German SoAS (with 5 items per subscale).

The subsequently performed two-factor model confirmatory factor analysis on the 10-item version of the German SoAS indicated a good model fit as well as good internal consistency of the two subscales. The two subscales showed only moderate negative correlations, indicating that even though they do share some variance, they still each measure a unique component of sense of agency. Further, by additionally performing a one-factor model confirmatory factor analysis we demonstrated that the one-factor model has a bad model fit. In spite of the good model fit of the two-factor model, it may be worthwhile to note that compared to all other items the factor loading of item 10 (“My behavior is planned by me from the very beginning to the very end.*“*) is rather low (<0.4). The content of item 10 differs slightly from that of the other items, as it captures action planning rather than performing an action. However, because action plans/intentions are an important aspect in sense of agency (*cf.* Pacherie, 2007), we decided to retain this item in the scale due to its theoretical relevance.

Taken together, the present results demonstrate that SoPA and SoNA are two distinct factors (rather than two facets of the same construct) and strongly support the assumption of the multidimensional nature of general agency beliefs (as already proposed by Tapal et al., 2017 for the original Hebrew version). As outlined in the introduction, theoretical support for this distinction between SoPA and SoNA is provided by other studies, which observed that feelings of helplessness and feelings of control are only moderately (negatively) correlated (Karsh et al., 2018) and that the feeling of being an agent and the feeling of not being an agent may be associated with different neuronal structures (Farrer and Frith, 2002; Farrer et al., 2003; see also Tapal et al., 2017 for an extensive discussion on this topic).

Similar to Tapal et al. (2017) (see Table 2), the analysis of the relation between general agency beliefs, as assessed with the German SoAS, and other, similar constructs, revealed significant positive correlations for SoPA with internal locus of control and self-efficacy, as well as a significant negative correlation with external locus of control. The opposite data pattern was observed for the SoNA subscale. It is not surprising that locus of control and general self-efficacy are related to one’s general agency beliefs, as all of those constructs capture overlapping aspects of control beliefs. Nevertheless, the correlations were only of moderate height, indicating that the constructs are still distinct enough from each other (e.g., general self-efficacy captures control beliefs related to goal related actions, whereas general agency beliefs capture control beliefs dissociated from goal attainment, see also Tapal et al., 2017). We further observed positive correlations for SoPA with positive body image (vital body dynamics) and with some of the subscales measuring interoceptive awareness as well as a significant negative correlation with negative body image (rejecting body evaluation). Again, the opposite data pattern was observed for the SoNA subscale. Sense of agency and sense of body ownership (feeling that this body part is one’s own) both require metacognitive monitoring of bodily states. Hence it is not surprising that general agency beliefs are related to aspects of body consciousness like body image and interoceptive awareness. However, similar as in the original Hebrew version of the SoAS (Tapal et al., 2017), the observed correlations were mostly low, whereas the correlations with locus of control and self-efficacy were of moderate height. This was to be expected. Even though sense of body ownership often shares aspects with sense of agency, it is still a more distinct concept than locus of control and self-efficacy, as it does not directly address control beliefs.

Additionally, corresponding to Tapal et al. (2017), we observed that the free will subscale was positively correlated with SoPA and negatively correlated with SoNA. This finding corroborates previous studies, indicating that sense of agency forms an important building block for beliefs in free will, and that, conversely, strengthening one’s free will beliefs can enhance sense of agency (Aarts and van den Bos, 2011; Lynn et al., 2014). Correlations were only of moderate height, lending support to the conceptual distinction between general agency beliefs and free will. At the same time, the low correlations with the subscale indeterminism/change as well as the nonsignificant correlations with the subscales biological determinism and incompatibilism indicate that one’s general agency beliefs are conceptually quite different from more philosophical notions of determinism. Presumably, because even if one believes on a more abstract or philosophical level that, for instance, certain biological factors determine one’s behavior, they may still feel in control of their own everyday life actions. Taken together, the pattern of low to moderate correlations between the SoAS and other, related constructs indicates that despite some overlap between certain aspects of the constructs, the two SoAS subscales are conceptually distinct enough from other constructs to warrant their uniqueness, thereby supporting construct validity of the German SoAS.

We investigated incremental validity of the German SoAS, by testing whether the German SoAS can account for additional variance in the extent of subclinical symptoms of certain mental disorders, beyond variance explained by conceptually similar constructs. Our results indicate that general self-efficacy is a negative predictor for the extent of subclinical symptoms of obsessive-compulsive disorder, depressive disorder, and schizotypal personality disorder. Similarly, internal locus of control is a negative predictor for the extent of subclinical symptoms of depressive disorder, whereas external locus of control is a positive predictor for the extent of subclinical symptoms of obsessive-compulsive disorder and depressive disorder. This seems reasonable as it has previously been observed that general self-efficacy and locus of control are related to mental health issues (e.g., Benassi et al., 1988; Kim, 2003; Kalafat et al., 2010). More importantly, the present results indicate that the German SoAS (in particular, the SoNA subscale) accounts for additional variance in the extent of subclinical symptoms of schizotypal personality disorder, over and above locus of control and general self-efficacy. However, the explained variance is rather low. Further, evidence regarding an incremental value of the SoAS in explaining variance of subclinical symptoms of obsessive-compulsive disorder was weak (Bayesian analysis) or not apparent (frequentist analysis, contradicting the results of Tapal et al., 2017, see Table 2). Further, we did not observe evidence for an incremental value of the SoAS predicting subclinical symptoms of depressive disorder (in accordance with the results of Tapal et al., 2017, Bayesian analysis even indicated substantial evidence against an incremental value of the SoAS). Mental health issues are associated with many predictors including but not limited to genetic risk factors, social support, and personality traits (Quilty et al., 2009; McGrath et al., 2013; Wang et al., 2018). Thus, it is not surprising that general agency beliefs only play a subordinate role. This may be further corroborated by the fact that we did not investigate clinical populations. The association between general agency beliefs and symptoms of mental disorders may be more pronounced in clinical samples. Nevertheless, results regarding schizotypal personality disorder and obsessive-compulsive disorder are promising and provide tentative evidence for the incremental validity of the German SoAS, which however should be investigated more thoroughly in follow-up studies.

Even though most psychometric properties of the German SoAS are satisfying, the test–retest reliability gives some cause for concern. For both the SoPA and SoNA agency subscales values were 0.7, which demonstrates a still acceptable, but rather moderate test-rest reliability (Koo and Li, 2016), whereas reliability coefficients in the original Hebrew version (Tapal et al., 2017) and the French translation (Hurault et al., 2020) were considered as good (see Table 1). One explanation may be that data collection for the present study took place in the midst of the Covid-19 pandemic. This may have impacted many people’s sense of agency (beliefs) differently at different times, as sometimes even basic everyday life actions such as leaving the house or meeting friends was outside of one’s control (Dhillon and Mishra, 2020). One may speculate that these special circumstances may have affected one’s general agency beliefs, in particular resulting in changes in one’s general agency beliefs over time and thus in a reduced re-test reliability. Another, more methodological, explanation may be that test–retest reliability was assessed on a relatively small subsample, as the response rate to the follow-up survey was rather low (*cf.* Kennedy, 2022 for the effect of sample size on test–retest reliability). As the claim that the SoAS measures one’s general, context-free agency beliefs may be questioned if test–retest reliability is not strong enough, it seems vital for future studies to investigate test–retest reliability of the German SoAS more thoroughly and to assess whether one’s general, context-free agency beliefs are malleable (e.g., due to major life events). Nevertheless, it is important to point out the values in the present study are still considered as acceptable indicating that the German SoAS allows to measure people’s cross-situational, general agency beliefs.

A limitation of the present study, as well as for the other versions of the SoAS (*cf.* Tapal et al., 2017; Hurault et al., 2020) is that we only assessed test–retest reliability after a time period of 2 months, i.e., short-term test–retest reliability. Thus, long-term test–retest reliability of the SoAS still remains unclear so far. One may also consider it a limitation that we simultaneously collected data to assess factorial validity and construct validity. Thus, one may argue that construct validity was not assessed with the “final” German SoAS (which does not include item 4). However, we believe that it is unlikely that exclusion of this item would have influenced the response behavior toward the other items. A further limitation of the present study is that we did not directly translate the validated Hebrew items into German, but instead translated the English items provided by the authors (Tapal et al., 2017). However, one may argue that the English scale is the “original” scale (even though this version was never validated) as Tapal et al. (2017) originally generated their items in English and afterwards used a translation/backtranslation approach to translate them into Hebrew. Further, even though the English scale was never thoroughly validated, it has already been used in other studies, which could replicate the two-factor structure and observed good internal consistency for both subscales (Cronbach’s α for the SoPA: 0.79, Cronbach’s α for the SoNA: 0.84, *cf.* Skeba et al., 2021). Thus, there is tentative evidence that the English items are suitable to assess one’s general, context-free agency beliefs. Nevertheless, a validity study of the English items with an English-speaking sample would reinforce the results of our study (see also Hurault et al., 2020 for such an argument regarding the French version). Another limitation is that, similar to Tapal et al. (2017), we only validated the German SoAS in a sample of rather young and more female than male participants and thus could not assess measurement invariance (i.e., whether participants across different groups interpret the content of each item in the same way; Schmitt and Kuljanin, 2008). Thus, future studies may investigate whether the measurement model of the German SoAS obtained in the present study holds true across subsamples differing for instance in gender (see also Hurault et al., 2020 who observed measurement invariance across gender for the French SoAS), age, or ethnicity.

Additionally, future studies should systematically investigate how general, context-free agency beliefs assessed via the SoAS relates to different contextualized, situation-specific experiences of agency, as assessed with either direct agency judgments such as self-recognition judgments (e.g., Aarts et al., 2005) or control judgments (e.g., Wenke et al., 2010), or with indirect SoA measures such as intentional binding (*cf.* Haggard et al., 2002). Further, the German SoAS may be used to investigate the role of general agency beliefs in different clinical populations suffering for instance from depression, posttraumatic stress disease, or schizophrenia. For instance, it may be of interest to investigate whether reduced general agency beliefs constitute a risk factor for developing certain mental disorders.

In conclusion, the present study provides a German version of the SoAS, for which the two-factorial structure (SoPA and SoNA) of the original Hebrew version (Tapal et al., 2017) was replicated. The German SoAS shows good psychometric properties (i.e., good model fits, good internal consistency) and moderate test–retest reliability. Additionally, similar to the original Hebrew version of the SoAS (Tapal et al., 2017), evidence for construct validity and tentative evidence for incremental validity was provided. Taken together, this indicates that the German SoAS is a valid and suitable instrument to assess one’s general, context-free agency beliefs.

## Data availability statement

The datasets presented in this study can be found in online repositories. The names of the repository/repositories and accession number(s) can be found at: https://osf.io/muhzr/.

## Ethics statement

The studies involving humans were approved by Research Committee for Scientific Ethical Questions (RCSEQ). The studies were conducted in accordance with the local legislation and institutional requirements. The participants provided their written informed consent to participate in this study.

## Author contributions

VB, DW, and MR contributed to designing the research and translating the sense of agency scale into German. VB programmed the online survey and supervised data collection and wrote the first draft of the manuscript. VB and MR contributed to analyzing the data. MR and DW gave feedback on the manuscript. All authors contributed to the article and approved the submitted version.

## Funding

This work was financially supported by a grant from the Tyrolean Science Fund (TWF): F.16581/5-2019 to VB.

## Conflict of interest

The authors declare that the research was conducted in the absence of any commercial or financial relationships that could be construed as a potential conflict of interest.

## Publisher’s note

All claims expressed in this article are solely those of the authors and do not necessarily represent those of their affiliated organizations, or those of the publisher, the editors and the reviewers. Any product that may be evaluated in this article, or claim that may be made by its manufacturer, is not guaranteed or endorsed by the publisher.

## Footnotes

^1^Note that even though we assessed the same constructs as Tapal et al. (2017), we used different questionnaires to assess those constructs, because we used questionnaires that were already validated in German language.

^2^ https://www.jamovi.org/

^3^ https://webpower.psychstat.org

^4^ https://cran.r-project.org

^5^ https://jasp-stats.org/

## Appendix

German version of the sense of agency scale (German SoAS).

Instruktion:

Bitte geben Sie an, wie sehr Sie den folgenden Aussagen zustimmen.

Antwortskala:

siebenstufige Ratingskala von (1) stimme überhaupt nicht zu bis (7) stimme vollkommen zu.

Subskalen:

- Positives Sense of Agency (SoPA): Item 1, 6, 7, 10, 11
- Negatives Sense of Agency (SoNA): Item 2, 3, 5, 8, 9

Items:

1. Ich habe die volle Kontrolle darüber, was ich tue.
2. Ich bin nur ein Instrument in den Händen von jemandem oder von etwas anderem.
3. Meine Handlungen geschehen einfach ohne meine Absicht.
4. Meine Bewegungen geschehen automatisch - mein Körper macht sie einfach.*
5. Im Allgemeinen überraschen mich die Ergebnisse meiner Handlungen.
6. Sachen, die ich mache, unterliegen nur meinem freien Willen.
7. Die Entscheidung, ob und wann ich handle, liegt in meinen Händen.
8. Nichts was ich mache, ist wirklich freiwillig.
9. Während ich handle, fühle ich mich wie ein ferngesteuerter Roboter.
10. Mein Verhalten ist von mir von Anfang bis Ende durchgeplant.
11. Ich bin vollkommen verantwortlich für alles, was sich aus meinen Handlungen ergibt.

*Please note: Item 4 is not included in the final German version of the SoAS as it did not fulfill all item inclusion criteria (the factor loading was less than 0.4 on the primary factor) and thus cannot be unambiguously assigned to one factor.
