# Supplementary material for: A German translation and validation of the sense of agency scale
Source: Front Psychol. 2023 Sep 14;14:1199648. doi: 10.3389/fpsyg.2023.1199648 (PMC10539649; doi:10.3389/fpsyg.2023.1199648)
Supplement: Supplementary file 1 [file Table_1.doc]

SUPPLEMENTAL MATERIAL

**A German translation and validation of the sense of agency scale**

**Victoria K. E. Bart^1^, Dorit Wenke^2^, & Martina Rieger^1^**

1 Institute of Psychology, UMIT TIROL - Private University for Health Sciences and Health Technology, Hall in Tyrol. Austria

2 Department of Psychology, PFH Private University of Applied Sciences, Göttingen, Germany

|  | 1 | 2 | 3 | 4 | 5 | 6 | 7 | 8 | 9 | 10 | 11 | 12 | 13 | 14 | 15 |
| --- | --- | --- | --- | --- | --- | --- | --- | --- | --- | --- | --- | --- | --- | --- | --- |
| **Locus of control** |  |  |  |  |  |  |  |  |  |  |  |  |  |  |  |
| 1. Internal locus of control | ----------- |  |  |  |  |  |  |  |  |  |  |  |  |  |  |
| 2. External locus of control | -.35*** | ---------- |  |  |  |  |  |  |  |  |  |  |  |  |  |
| **3. General self-efficacy** | .43*** | -.32*** | --------- |  |  |  |  |  |  |  |  |  |  |  |  |
| **Belief in free will** |  |  |  |  |  |  |  |  |  |  |  |  |  |  |  |
| 4. Situational determinism | .14** | .001 | .12** | --------- |  |  |  |  |  |  |  |  |  |  |  |
| 5. Free will | .28*** | -.27*** | .28*** | .07 | --------- |  |  |  |  |  |  |  |  |  |  |
| 6. Indeterminism/change | -.07 | .17*** | -.07 | .14** | -.05 | --------- |  |  |  |  |  |  |  |  |  |
| 7. Biological determinism | .06 | .06 | .06 | .30*** | .03 | .27*** | --------- |  |  |  |  |  |  |  |  |
| 8. Incompatibilism | -.01 | .04 | .01 | .17*** | .09 | .01 | .24*** | --------- |  |  |  |  |  |  |  |
| **Interoceptive awareness** |  |  |  |  |  |  |  |  |  |  |  |  |  |  |  |
| 9. Noticing | .17*** | -.06 | .13** | .004 | .15*** | -.17*** | .02 | -.002 | --------- |  |  |  |  |  |  |
| 10. Not-distracting | -.01 | -.07 | .01 | -.02 | -.08 | -.09* | -.09* | .001 | -.08 | --------- |  |  |  |  |  |
| 11. Not-worrying | .04 | -.13** | .17*** | -.09* | .06 | -.08 | -.20*** | -.08 | -.10* | .14** | --------- |  |  |  |  |
| 12. Attention regulation | .23*** | -.09* | .31*** | .12** | .18*** | -.07 | -.02 | .08 | .42*** | -.001 | .16*** | --------- |  |  |  |
| 13. Emotional awareness | .19*** | -.05 | .06 | .06 | .15*** | -.12** | .08 | .01 | .58*** | -.11* | -.12** | .41*** | --------- |  |  |
| 14. Self-regulation | .24*** | -.10* | .32*** | .17*** | .09* | -.08 | .05 | .05 | .43*** | .05 | .08 | .64*** | .48*** | --------- |  |
| 15. Body listening | .12** | .01 | .20*** | .13** | -.03 | -.10* | .06 | .04 | .45*** | .13** | -.02 | .52*** | .52*** | .69*** | --------- |
| 16. Trusting | .39*** | -.26*** | .38*** | .15*** | .18*** | -.11* | -.03 | .05 | .36*** | .04 | .11* | .45*** | .40*** | .48*** | .46*** |

**Table S1**. Results of Pearson correlations between the mean scores of locus of control (2 subscales: internal locus of control, external locus of control), general self-efficacy, free will (5 subscales: situational determinism, free will, indeterminism/change, biological determinism, incompatibilism), and interoceptive awareness (8 subscales: noticing, not-distracting, not-worrying, attention regulation, emotional awareness, self-regulation, body listening, trusting).

*Note.* * p < .05, ** p < .01, *** p < .001

**Table S2.** Results of Pearson correlations of the mean scores of the body image scales (2 subscales: rejecting body evaluation, vital body dynamics) with the mean scores of locus of control (2 subscales: internal locus of control, external locus of control), general self-efficacy, free will (5 subscales: situational determinism, free will, indeterminism/change, biological determinism, incompatibilism), and interoceptive awareness (8 subscales: noticing, not-distracting, not-worrying, attention regulation, emotional awareness, self-regulation, body listening, trusting).

|  | **Body image** | |
| --- | --- | --- |
|  | Rejecting body evaluation | Vital body dynamics |
| **Locus of control** |  |  |
| Internal locus of control | -.33*** | .39*** |
| External locus of control | .40*** | -.27*** |
| **General self-efficacy** | -.37*** | .51*** |
| **Belief in free will** |  |  |
| Situational determinism | -.01 | .07 |
| Free will | -.18*** | .17*** |
| Indeterminism/change | .15*** | .01 |
| Biological determinism | .16*** | .03 |
| Incompatibilism | .10* | -.02 |
| **Interoceptive awareness** |  |  |
| Noticing | -.12** | .19*** |
| Not-distracting | -.09* | .04 |
| Not-worrying | -.30*** | .12** |
| Attention regulation | -.21*** | .31*** |
| Emotional awareness | -.12** | .12** |
| Self-regulation | -.21*** | .30*** |
| Body listening | -.08 | .19*** |
| Trusting | -.56*** | .39*** |

*Note.* * p < .05, ** p < .01, *** p < .001,
The Pearson correlation between the two subscales of the body image scale (rejecting body evaluation, vital body dynamics) is *r* = -.37, *p* <.001.
